# Diverticule de l'urètre après urétro-méatoplastie: à propos d'un cas

**DOI:** 10.11604/pamj.2019.33.179.17406

**Published:** 2019-07-08

**Authors:** Mahdi Graiouid, Youness Chakir, Issam Jandou, Messian Gallouo, Yassine Larrache

**Affiliations:** 1 Service d'Urologie, CHU Ibn Rochd, Casablanca, Maroc

**Keywords:** Diverticule de l’urètre, accident de circoncision, urétroplastie, Urethral diverticulum, circumcision accident, urethroplasty

## Abstract

Nous rapportons l'observation d'un diverticule de l'urètre pénien chez un garçon de 14 ans survenu après une urétro et méatoplastie suite à un accident de circoncision. À travers cette observation et une revue de la littérature, nous décrirons les aspects diagnostiques et thérapeutiques des diverticules de l'urètre.

## Abstract (en)

We report the case of a 14-year old boy with penile urethral diverticulum occurred after urethromeatoplasty performed due to circumcision injury. This study and literature review aim to describe the diagnostic and therapeutic features of urethral diverticulum.

## Introduction

Les diverticules de l'urètre masculin sont des entités cliniques rares. Le diverticule posturétroplastie est une complication assez rare, peu décrite dans la littérature. Il peut être congénital ou acquis. La forme acquise est la plus fréquente représentant 90% des diverticules de l'urètre chez l'homme, le plus souvent compliquant une urétroplastie. Le traitement nécessitera une intervention à ciel ouvert qui consistera à réaliser une plastie de réduction.

## Patient et observation

**Figure 1:**
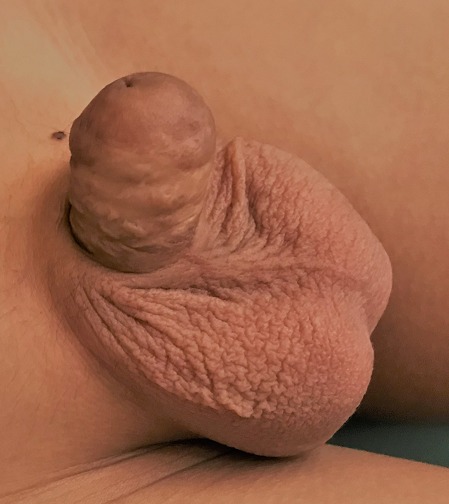
Aspect de la verge à l'examen clinique

**Figure 2:**
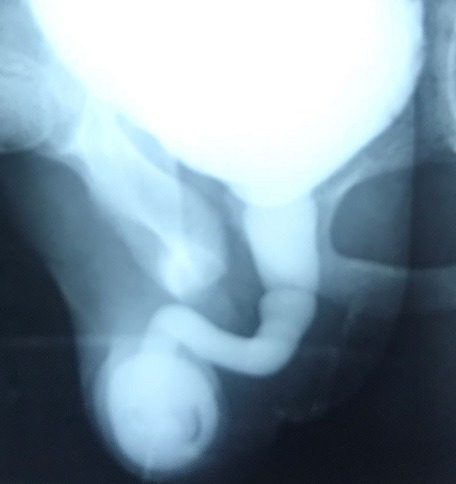
UCR objectivant l'image d'addition rétroméatique

Enfant de 14 ans, opéré à l'âge de 3 ans pour section subtotale du gland à la suite d'un accident de circoncision, il avait bénéficié d'urétro-méatoplastie avec des suites postop immédiates simples. Le patient a été perdu de vue puis consulte après onze ans pour une dysurie associée à une incontinence urinaire. L'examen urogénital met en évidence une verge de 5 cm, un méat apical réduit de calibre sans tuméfaction palpable (Figure 1). L'urétrocystographie mictionnelle après cathérisation difficile du méat urétral montre une image d'addition rétroméatique en rapport avec un diverticule et le reste de l'urètre est dilaté (Figure 2). L'échographie de la verge est normale. Le patient a été opéré et une mise à plat du diverticule et une excision de l'étoffe en excès ont été faites. Trois mois plus tard une plastie urétrale a été réalisée, associée à une méatotomie. L'évolution était bonne et aucune fistule ni récidive n'ont été constatées après 3 mois.

## Discussion

Le diverticule de l'urètre masculin est une entité rare, et aucun cas de diverticule de l'urètre chez l'enfant après réparation chirurgicale d'un accident de la verge n'a été rapporté dans la littérature [1]. Il peut être congénital ou acquis. La variété acquise est la plus fréquente des diverticules de l'urètre chez l'homme le plus souvent secondaire à une urétroplastie [2]. La symptomatologie clinique est polymorphe et dépend de l'existence ou non de complications. Le signe le plus constant est représenté par l'écoulement urinaire postmictionnel avec la présence de gouttes retardataires. Ils peuvent être révélés par des infections urinaires à répétition, hématurie, tuméfaction de la verge [3,4]. Le diverticule peut devenir volumineux ou se compliquer de calculs et se manifestera par une dysurie voire une rétention aiguë d'urines [5]. Le diagnostic du diverticule est confirmé par l'urétrocystographie rétrograde et mictionnelle qui apparaît comme une image d'addition, de forme ovalaire, le plus souvent au dépend de la face ventrale de l'urètre. Une sténose d'aval peut être retrouvée au niveau de l'anastomose distale de l'urètre. L'échographie peut compléter le bilan radiologique en visualisant le diverticule et les parois de l'urètre. Le traitement nécessitera une intervention à ciel ouvert. Il consiste à réaliser une excision du diverticule et un rétablissement de continuité en un temps. En cas de tissu péri-urétral inflammatoire ou une sténose d'aval, une mise à plat avec une plastie en 2^ème^ temps 3 mois plus tard est préférable [6,7].

## Conclusion

Le diverticule de l'urètre masculin après urétroplastie est une affection rare. Il doit être suspecté devant des troubles du bas appareil urinaire associés à une masse périurétrale. L'urétrocystographie est suffisante pour la confirmation du diagnostic. La diverticulectomie totale est le traitement de référence car il permet de rétablir une anatomie urétrale normale et d'éviter les complications.

## Conflits d’intérêts

Les auteurs ne déclarent aucun conflit d'intérêts.
